# PEDF-34 attenuates neurological deficit and suppresses astrocyte-dependent neuroinflammation by modulating astrocyte polarization via 67LR/JNK/STAT1 signaling pathway after subarachnoid hemorrhage in rats

**DOI:** 10.1186/s12974-024-03171-y

**Published:** 2024-07-21

**Authors:** Lei Wu, Yanchao Liu, Qiuguang He, Guangnan Ao, Ningbo Xu, Wangqing He, Xiao Liu, Lei Huang, Qian Yu, Hideki Kanamaru, Siyuan Dong, Shiyi Zhu, Ye Yuan, Mingyang Han, Yeping Ling, Lu Liu, Chenyu Wu, You Zhou, Prativa Sherchan, Jerry J. Flores, Jiping Tang, Xionghui Chen, Xuying He, John H. Zhang

**Affiliations:** 1 Department of Cerebrovascular Surgery, Neurosurgery Center, Engineering Technology Research Center of Education Ministry of China on Diagnosis and Treatment of Cerebrovascular Disease, Zhujiang Hospital, Southern Medical University, Guangzhou, 510280 Guangdong China; 2 Department of Physiology and Pharmacology, Basic Sciences, School of Medicine, Loma Linda University, Loma Linda, CA 92354 USA; 3 Department of Neurology, Guangdong Second Provincial General Hospital, Guangzhou, 510317 Guangdong China; 4 Department of Interventional Therapy, Zhujiang Hospital, Southern Medical University, Guangzhou, 510280 Guangdong China; 5 Department of Neurosurgery, Loma Linda University School of Medicine, Loma Linda, CA 92354 USA; 6 Department of Emergency Surgery, First Affiliated Hospital of Soochow University, Suzhou, 215000 Jiangsu China

**Keywords:** Subarachnoid hemorrhage, Non-integrin 67-kDa laminin receptor, Pigment epithelial-derived factor, Astrocyte polarization, Neuroinflammation

## Abstract

**Background:**

Reactive astrocytes participate in various pathophysiology after subarachnoid hemorrhage (SAH), including neuroinflammation, glymphatic–lymphatic system dysfunction, brain edema, BBB disruption, and cell death. Astrocytes transform into two new reactive phenotypes with changed morphology, altered gene expression, and secretion profiles, termed detrimental A1 and beneficial A2. This study investigates the effect of 67LR activation by PEDF-34, a PEDF peptide, on neuroinflammation and astrocyte polarization after the experimental SAH.

**Methods:**

A total of 318 male adult Sprague-Dawley rats were used in experiments in vivo, of which 272 rats were subjected to the endovascular perforation model of SAH and 46 rats underwent sham surgery. 67LR agonist (PEDF-34) was administrated intranasally 1 h after SAH. 67LR-specific inhibitor (NSC-47924) and STAT1 transcriptional activator (2-NP) were injected intracerebroventricularly 48 h before SAH. Short- and long-term neurological tests, brain water content, immunostaining, Nissl staining, western blot, and ELISA assay were performed. In experiments in vitro, primary astrocyte culture with hemoglobin (Hb) stimulation was used to mimic SAH. The expression of the PEDF-34/67LR signaling pathway and neuro-inflammatory cytokines were assessed using Western blot, ELISA, and immunohistochemistry assays both in vivo and in vitro.

**Results:**

Endogenous PEDF and 67LR expressions were significantly reduced at 6 h after SAH. 67LR was expressed in astrocytes and neurons. Intranasal administration of PEDF-34 significantly reduced brain water content, pro-inflammatory cytokines, and short-term and long-term neurological deficits after SAH. The ratio of *p*-JNK/JNK and *p*-STAT1/STAT1 and the expression of CFB and C3 (A1 astrocytes marker), significantly decreased after PEDF-34 treatment, along with fewer expression of TNF-α and IL-1β at 24 h after SAH. However, 2-NP (STAT1 transcriptional activator) and NSC-47924 (67LR inhibitor) reversed the protective effects of PEDF-34 in vivo and in vitro by promoting A1 astrocyte polarization with increased inflammatory cytokines.

**Conclusion:**

PEDF-34 activated 67LR, attenuating neuroinflammation and inhibiting astrocyte A1 polarization partly via the JNK/STAT1 pathway, suggesting that PEDF-34 might be a potential treatment for SAH patients.

**Graphical Abstract:**

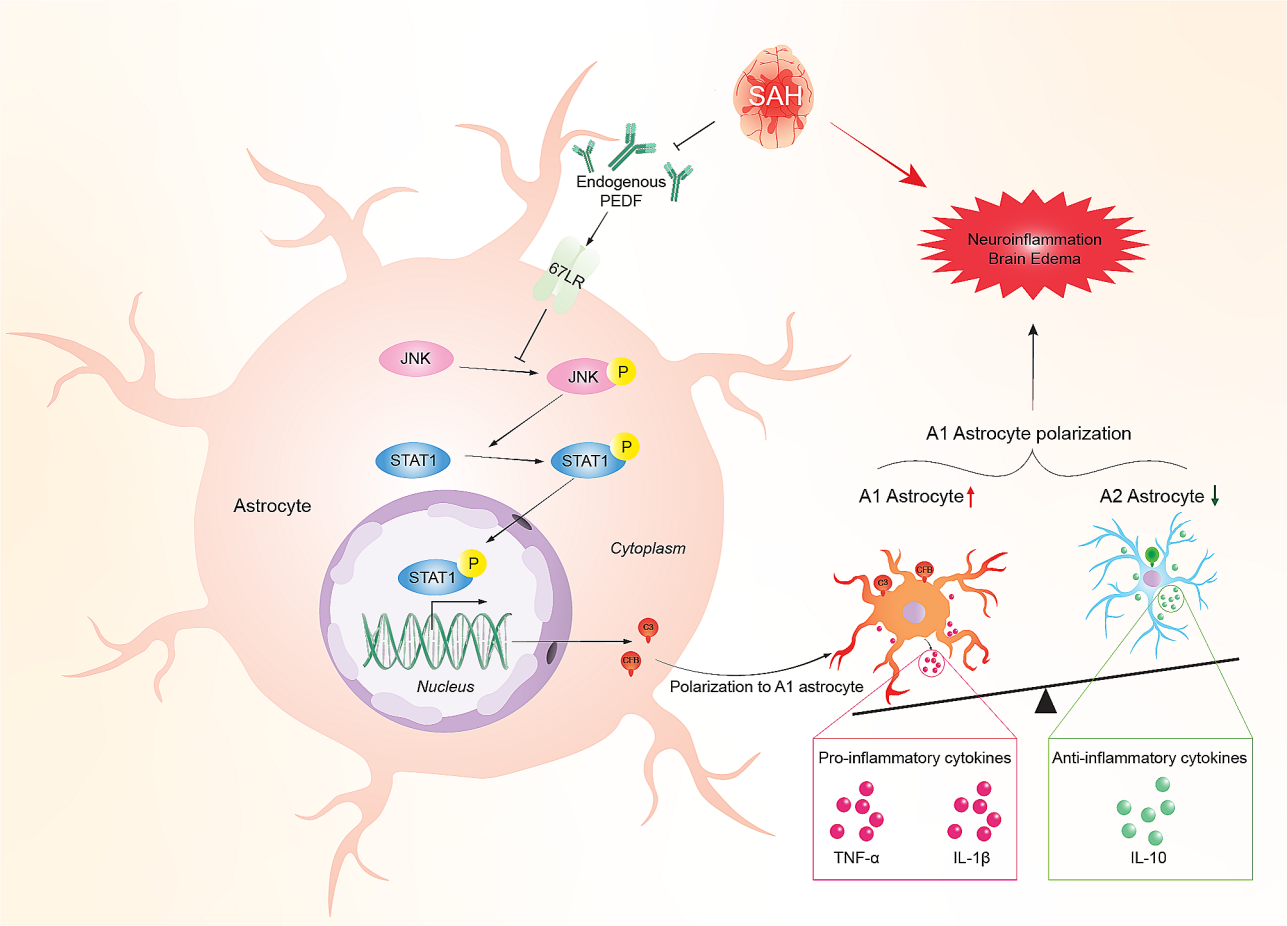

**Supplementary Information:**

The online version contains supplementary material available at 10.1186/s12974-024-03171-y.

## Introduction

Subarachnoid hemorrhage (SAH) is a life-threatening neurovascular disease with a 25–50% fatality rate and high disability [1–3]. Early brain injury (EBI) is the primary cause of mortality in SAH patients [4], which consists of acute pathological processes occurring in the brain within the first 72 h after SAH. Neuroinflammation is attributed significantly to EBI pathogenesis. Therefore, anti-neuroinflammatory approaches would be beneficial in treating SAH.

Pigment epithelial-derived factor (PEDF), a 50-kDa monomeric glycoprotein and member of the serine protease inhibitor superfamily [5], protects cells from oxidative stress [6]. Previous studies demonstrated the anti-inflammation properties of PEDF [7–12] which effectively inhibited expressions of inflammatory cytokines TNF-α and IL-1β in dry eye disease [8, 13]. By activating the non-integrin 67-kDa laminin receptor (67LR), PEDF reduced myocardial infarct size and vascular leakage in rats [10]. 67LR is involved in cell adherence to the basement membrane and regulates the interactions between laminin and other receptors [14]. The laminin receptor activation inhibits endothelial tissue factor expression by inhibiting JNK phosphorylation [15], thus inhibiting the phosphorylation and transcription of STAT1 [3, 16, 17]. In addition, PEDF-peptide was shown to upregulate AQP4 by activating 67LR in astrocytes, maintaining the integrity of the blood-brain barrier (BBB) [18, 19]. However, the effects of 67LR activation by PEDF in EBI after SAH have never been reported.

Astrocytes are important in maintaining normal BBB structures and functions in the central nervous system (CNS). Upon being stimulated by various inflammatory cytokines after SAH, resting astrocytes become reactive and display two phenotypes, A1 and A2 [20, 21]. A1 astrocytes, marked with complement factor B (CFB) or complement component 3 (C3), cause cell death by releasing pro-inflammatory factors, while A2 astrocytes, marked with S100A10, perform neuroprotective effects by secreting anti-inflammatory mediators [22]. A1 astrocytes were prevalent and persistent relative to A2 astrocytes in EBI after SAH, contributing to neuroinflammation and neuronal death [23–25]. Prokineticin 2 has been shown to promote astrocytic polarization to an A2 phenotype and reduce SAH-induced neuronal injury and behavior dysfunction [23]. Furthermore, inhibiting transformation into A1 astrocytes would attenuate neuronal death by reducing the release of pro-inflammatory factors in EBI after SAH [25].

In the present study, the effects of PEDF/67LR signaling on astrocyte polarization and neuroinflammation were investigated in experimental SAH. Considering the therapeutic limitations of full-length PEDF, including its short half-life, unstable pharmacological activity, and delivery pathway [26], PEDF-34, a peptide fragment of PEDF, was used [27]. The hypothesis was that activating 67LR by PEDF-34 would attenuate neuroinflammation and downregulate A1 astrocyte polarization via inhibiting the JNK/STAT1 pathway after SAH.

## Materials and methods

### Animals

All experimental procedures were approved by the Institutional Animal Care and Use Committee (IACUC) of Loma Linda University (#23 − 004) following the National Institutes of Health Guidelines for the Care and Use of Animals in Neuroscience Research and ARRIVE guidelines. The study utilized 318 male Sprague-Dawley rats (weighing 270–320 g, Envigo, Indianapolis, IN, USA), and the specific rat usage is presented (Additional file: Figure S1). Animals were housed in a controlled environment maintained at a constant temperature of 25 ± 1 °C and relative humidity of 60 ± 5% and had access to food and water on a 12-hour light-dark cycle.

### SAH model

A previously published endovascular perforation SAH model was used [28]. Briefly, rats were intubated and mechanically ventilated with 2–3% isoflurane in the air, maintaining a respiratory rate of 77 throughout the operation (isoflurane was reduced to 1.5% after puncture). A sharp 4 − 0 monofilament polypropylene suture was inserted from the external carotid artery and common carotid bifurcation into the left internal carotid artery. The nylon suture was advanced until encountering resistance at the bifurcation of the anterior and middle cerebral arteries, then pushed further to puncture the blood vessels before being immediately withdrawn. The rats in the Sham group underwent the same process above but without vessel puncture. The skin incision was sutured after surgery, and the rats were kept on a heating pad at 37.5 °C. Continuously monitor the vital signs of rats until they fully recover.

### Assessment of SAH grade

The SAH grade was evaluated according to the distribution of blood and blood clots in the six parts of the basal brain cistern. Each part was considered by a score from 0 to 3 in terms of the amount of subarachnoid blood and blood clots present [29, 30]. The mildly injured rats with a score of 8 or less at 24 h after SAH were excluded [30, 31]. The assessment was done by a researcher who was blind to the information of experimental groups.

### Cell culture and SAH model in vitro

Primary astrocyte culture and hemoglobin stimulation were used as an in vitro model of SAH. Primary astrocytes were extracted from P2 Sprague–Dawley rat pups. Briefly, 10 ml of HBSS (14,175,095, Gibco, Carlsbad, USA) was added to the culture dishes. After anesthesia and sterilization, the pups’ heads were cut off and put into HBSS. Whole brain tissues were removed from the pup’s heads, and pia matter was carefully separated from the brains. Cortex tissues were collected, transferred to a centrifuge tube, and lysed with 0.25% EDTA-trypsin (25,200,072, Gibco, Carlsbad, USA) at 37 °C for 15 min. Then, the lysing was terminated by a solution containing 79% high glucose DMEM (11,965,092, Gibco, Carlsbad, USA), 20% FBS (A5256701, Gibco, Carlsbad, USA), and 1% Antibiotic-Antimycotic (100×, 15,240,062, Gibco, Carlsbad, USA). After centrifuging at 1000 rpm for 5 min, the cells were re-suspended with astrocyte culture medium, which contained 88% high glucose DMEM, 10% FBS, 1% Antibiotic-Antimycotic and 1% GlutaMAX™ Supplement (35,050,061, Gibco, Carlsbad, USA). The cells were transferred to dishes and cultured in an incubator at 37 °C, with 5% CO2. The medium was half changed every 4 days. After 7–8 days of culture, astrocytes were subjected to in vitro experiments.

For Hemoglobin stimulation, rat primary astrocytes were seeded onto 6-well plates at a density of 2 × 10^5^, and hemoglobin (9008-02-0, Sigma, USA) was dissolved by PBS at a concentration of 250µM (stock solution) (161 mg Hemoglobin + 10 ml PBS). After culture maturations, the medium was completely replaced with a normal medium supplemented with a final concentration of 25µM Hemoglobin (0.2 ml Hemoglobin stock solution + 1.98 ml normal medium).

### Experimental design

The supplementary file (Additional file: Figures S1 and S2) shows the experimental design and the distribution of animals according to different groups.

#### Experiment 1: time course of PEDF, 67LR, *p*-JNK, JNK, *p*-STAT1, STAT1, CFB, C3, S100A10 and cellular localization of 67LR in SAH rat

Forty-two rats were randomly assigned to seven groups (*n* = 6/group): Sham group and six SAH groups (3 h, 6 h, 12 h, 24 h, 48 h, and 72 h after SAH). Western blot analysis was used to quantify expression changes of PEDF, 67LR, *p*-JNK, JNK, *p*-STAT1, STAT1, CFB, C3, and S100A10. An additional 8 rats were randomly assigned to the Sham (*n* = 4) and SAH group (24 h, *n* = 4) to evaluate cellular localization of 67LR using double immunofluorescence staining.

#### Experiment 2: the effect of 67LR activation by PEDF-34 on short-term neurological outcomes after SAH

Thirty rats were randomly assigned to five groups (*n* = 6/group): Sham, SAH + Vehicle, SAH + PEDF-34 (1 µg/kg), SAH + PEDF-34 (3 µg/kg), and SAH + PEDF-34 (9 µg/kg). Modified Garcia score, beam balance, and brain water content assessments were conducted 24 h after SAH. Based on the short-term neurological outcomes, the best dose of PEDF-34 (3 µg /kg) was selected for the following experiments, including long-term neurological outcomes and anti-neuroinflammation mechanism. Additional 12 rats were randomly divided into three groups (*n* = 4/group), Sham, SAH + Vehicle, and SAH + PEDF-34 (3 µg/kg), to assess expressions of IL-1β and IL-10 in the ipsilateral hemisphere at 24 h after SAH using double-immunofluorescence staining.

#### Experiment 3: the effect of PEDF-34 on long-term neurological outcomes after SAH

Thirty rats were randomly assigned to three groups (*n* = 10/group): Sham, SAH + Vehicle, SAH + PEDF-34 (3 µg /kg). The Rotarod test was conducted on days 7, 14, and 21 after SAH. Morris water maze was performed on days 23–27 after SAH. The rats were euthanized on day 28, and Nissl staining was performed to assess brain neuronal degeneration.

#### Experiment 4: dosage testing of NSC-47924 and 2-NP in SAH rat model

For the dose test of the 67LR inhibitor, NSC-47924, and STAT1 transcriptional activator (2-NP), 60 rats were randomly divided into ten groups. Western blotting was conducted to measure the expression of the 67LR and its downstream *p*-JNK, JNK, *p*-STAT1, and STAT1 expression.

#### Experiment 5: in vivo mechanism study (67LR/JNK/STAT1 signaling pathway)

Thirty-six rats were randomly divided into six groups (*n* = 6/group): Sham, SAH + Vehicle, SAH + PEDF-34 (3 µg/kg), SAH + PEDF-34 (3 µg/kg) + Vehicle, SAH + PEDF-34 (3 µg/kg) + NSC-47924 (50 µg/kg), and SAH + PEDF-34 (3 µg/kg) + STAT1 transcriptional activator (2-NP, 15 µg/kg). The expressions of 67LR, *p*-JNK, JNK, *p*-STAT1, STAT1, CFB, C3, and S100A10 were evaluated by Western blot assay. 36 rats were randomly divided into the same six groups for ELISA assay as the Western blotting experiments group (*n* = 6/group). The ipsilateral brain hemisphere was collected to measure the cytokine level, including TNF-α, IL-1β, and IL-10. Another set of 24 rats was divided randomly into the same 6 groups (*n* = 4/group); immunofluorescence staining was performed to assess astrocyte polarization by co-staining of C3 (A1 marker) or S100A10 (A2 marker) with GFAP (astrocyte marker).

#### Experiment 6: in vitro mechanism study (primary astrocyte culture)

In an in vitro experiment, primary astrocyte culture was used, and SAH was mimicked by hemoglobin (Hb) stimulation. Knockdown efficacies were measured by western blot after transfection with different MOIs of sh-67LR (MOI: 1:1, 3:1, 10:1). Cell viability was conducted by CCK-8 assay after treatment with different doses of 2-NP (13.5µM, 45µM, 135µM). Cultured cells were randomly divided into six groups: Sham, Hb + Vehicle, Hb + PEDF-34, Hb + PEDF-34 + 67LR sh-control (MOI 3:1), Hb + PEDF-34 + sh-67LR (MOI 3:1), Hb + PEDF-34 + 2-NP (13.5 µΜ). Western blotting was performed to measure the expression of 67LR, *p*-JNK, JNK, *p*-STAT1, STAT1, CFB, C3, and S100A10 levels (*n* = 6/group). ELISA was performed to measure the cytokines level, including TNF-α, IL-1β, and IL-10 (*n* = 6/group). Astrocyte polarization was assessed by co-localization of C3 (A1 marker) or S100A10 (A2 marker) with GFAP by immunofluorescence staining (*n* = 4/group).

### Drug administration

In in vivo experiments, a vehicle (10% DMSO) or PEDF-34 (1 µg/kg, 3 µg/kg, or 9 µg/kg) was administrated intranasally 1 h after SAH. For the short-term neurological outcomes and mechanism study, PEDF-34 was administered at 1 h post-SAH, once daily for 7 days after SAH for long-term neurological tests. The 67LR inhibitor (NSC-47924, 50 µg/kg, Focus Biomolecules, USA) and the STAT1 transcriptional activator (2-(1,8-naphthyridin-2-yl) phenol, 2-NP; 15 µg/kg, ab142704, Abcam, Cambridge, USA) were administrated intracerebroventricularly (i.c.v.) 48 h before SAH. For i.c.v injection in isoflurane-anesthetized rats, a 10 µl Hamilton syringe (Microliter 701, Hamilton Company, USA) was inserted through a burr hole on the skull into the left lateral ventricle (0.9 mm posterior and 1.5 mm lateral to bregma, 3.2 mm depth). The rate of i.c.v injection was controlled at 1 µl/min using an infusion pump. After injection, the needle was kept in situ for 5 min and then slowly retracted. The burr hole was sealed with bone wax immediately after removal of the needle. The rats were kept on a warm blanket to recover from anesthesia.

In in vitro experiments, PEDF-34 (10.8 pM) [32] was administrated 1 h after Hb stimulation. STAT1 transcriptional activator (2-NP, 13.5 µM) was administrated 48 h before Hb stimulation. 67LR shRNA was administrated to culture medium 72 h before Hb stimulation. The vehicle and Hb groups were given the same dose of sh-control. 67LR shRNA and sh-control were customized and purchased from Horizon Discovery Biosciences Limited (V3SR7594-237950775, Horizon Discovery Biosciences Limited, Cambridge, UK). The sequence is listed as follows: sh-67LR: 5′- GGGTTCTCAATGGCAACAA-3′; sh-control, 5′-GCATATGTGCGTACCTAGCAT-3′. The virus titer of shRNA was 1.0 × 10^8^ TU/ml. The western blot result of the MOI test indicated that 3:1 is performing the best transfection ability (Additional file: Figure S3B). The STAT1 transcriptional activator (2-NP) dose test was performed [33] (Additional file: Figure S3E, F).

### **Short-term neurological performance evaluation**

The modified Garcia scale consists of six parts with 0–3 points for each part: judgments of spontaneous activity, symmetry in the movement of four limbs, forepaw outstretching, climbing, body proprioception, and response to vibrissae touch. In the beam balance test, the walking distance on a beam was evaluated within 1 min using a score of 0–4 (a higher score indicates a longer walking distance).

### Long-term neurological performance evaluation

#### Rotarod test

As described previously [14], the Rotarod test assessed motor coordination and balance by measuring the time staying on a rotating rod on days 7, 14, and 21 after SAH. The rotating speed was started at 5 RPM or 10 RPM and increased by 2 RPM every 5 s. A photo beam circuit recorded how long rats could stay on the accelerating rotating cylinder.

### Morris water maze

The water maze test was initiated between days 22 and 27 to assess the spatial learning capacity and memory. As previously demonstrated [34], a cue water maze test was conducted on day 22 followed by spatial water maze test on 23–26 days after SAH. On day 27, the probe test was performed, and the animals were placed in the pool without the escaping platform. The swim speed and path of the animals were evaluated using the video tracking system SMART-2000 (Noldus Ethovision; Noldus, Tacoma, USA).

### Brain water content

The brain water content (BWC) assessment was consistent with 4 parts of the rat’s brain: the left brain, right brain, cerebellum, and brain stem. Each part was weighed as its wet weight and then obtained its constant dry weight by drying in a 105 °C oven for 72 h. The percentage of BWC was calculated using the formula: [(wet weight - dry weight)/wet weight] × 100%.

### Immunofluorescence (IF)

In in vivo experiments, anesthetized animals were euthanized with cold 10% formalin transcranial perfusion at various time points after SAH or sham surgery (see study design). The brains were post-fixed in 10% formalin at 4°C for 24 h, followed by dehydration in 70% sucrose for another 72 h After the brain was extracted and embedded in Scigen Tissue-Plus™ O.C.T. Compound (23-730-571, Thermo Fisher Scientific, Carlsbad, USA), brain sections were cut into 10 µm coronal slices in a cryostat (LM3050S, Leica Microsystems, Bannockburn, Germany) and mounted on adhesive glass slides. For IF staining, slides were washed by 1× PBS three times and incubated in 0.1% Triton-X-100 (Thermo Fisher Scientific, Carlsbad, USA) for 30 min at 4° C. After adding 5% donkey serum to block for 1 h at a room temperature, the slides were incubated with the primary antibodies overnight at 4° C. The primary antibodies were C3 (1:100, ab200999, Abcam, Cambridge, USA), S100A10 (1:100, PA5-95505, Thermo Fisher Scientific, Carlsbad, USA) and 67LR (1:100, ab133645, Abcam, Cambridge, USA), GFAP (1:200, ab279289 Abcam, Cambridge, USA), NeuN (1:200, ab177487, Abcam, Cambridge, USA), and Iba1. (1:200, ab178846, Abcam, Cambridge, USA). On the second day, slides were washed with 1× PBS three times and incubated with the second antibody for 1 h at room temperature. The slides were washed with 1× PBS three times, and Aqueous Mounting Medium with DAPI (4’, 6-Diamidino-2-Phenylindole, Dihydrochloride, Jackson ImmunoResearch, West Grove, USA) was used before coverslips were applied. A fluorescence microscope (DMi8, Leica Microsystems, Germany) observed and photographed the slides.

In an in vitro experiment, the cultured primary astrocyte cells were fixed in 4% paraformaldehyde for 30 min at 48 h after Hb stimulation. After being blocked in 1% BSA for 1 h, the cells were incubated with primary antibodies against C3 (1:100, ab200999, Abcam, Cambridge, USA), S100A10 (1:100, PA5-95505, Thermo Fisher Scientific, Carlsbad, USA, ) or GFAP (1:500, ab279289 Abcam, Cambridge, USA) overnight at 4 °C. The next day, the cells were incubated with appropriate secondary fluorescent antibodies. Finally, cell nuclei underwent counterstaining of DAPI. The slides were observed and photographed using a confocal microscope (Leica, Wetzlar, Germany).

### ELISA

ELISA samples of brain tissue (left hemisphere) and cell cultures previously described [35]. TNF-α, IL-1β, and IL-10 were quantified using TNF alpha ELISA Kit (ab236712, Abcam, Cambridge, USA), IL-1β ELISA Kit (ab255730, Abcam, Cambridge, USA), and IL-10 ELISA Kit (ab214566, Abcam, Cambridge, USA) in accordance with the manufacturer’s instructions.

### Western blot analysis

The left ipsilateral hemisphere tissue was homogenized in RIPA lysis buffer (sc-24,948, Santa Cruz Biotechnology, USA) with protease inhibitor cocktail for 15 min and then centrifuged at 14,000 g at 4 °C for 30 min. After collecting the supernatant, protein quantification was performed using a detergent-compatible assay (Bio-Rad, DC™ Protein Assay). Western blotting was performed as described previously [36]. The primary antibodies included the Anti-PEDF (1:1000, ab307083, Abcam, Cambridge, USA), Anti-67LR antibody (1:1500, ab133645, Abcam, Cambridge, USA), Anti-*p*-JNK antibody (1:2000, ab131499, Abcam, Cambridge, USA), Anti-JNK antibody (ab179461, Abcam, Cambridge, USA), Anti-*p*-STAT1 antibody (1:1000, sc-8394, Santa Cruz Biotechnology Inc., USA), Anti-STAT1 antibody(1:1000, sc-464, Santa Cruz Biotechnology Inc., USA), Anti-CFB antibody (1:1500, ab231072, Abcam, Cambridge, USA), Anti-C3/C3b antibody (1:1000, ab200999, Abcam, Cambridge, USA), Anti-S100A10 antibody (1:1000, PA5-95505, Thermo Fisher Scientific, Carlsbad, USA), and Anti-β-actin (1:1000, ab8227, Abcam, Cambridge, USA).

### Statistical analysis

All analyses were performed and graphed using GraphPad Prism 10.0 software (GraphPad Software, La Jolla, CA, USA). The data were presented as mean ± standard deviation (means ± SD). A one-way analysis of variance (ANOVA) followed by Tukey’s post hoc test was used to test the differences among three or more groups. Long-term neurobehavioral tests were analyzed using Two-way ANOVA. Differences were considered significant when *p* < 0.05.

### Result

#### Mortality and SAH grade

A total of 318 rats were used, of which 46 rats underwent sham surgery, and 272 rats underwent SAH induction. Of the 272 SAH rats, 9 were excluded from this study due to low SAH grading scores. The overall mortality of SAH rats was 11.79% (31/263). No rats in the sham group died. Subarachnoid blood clots were around the circle of Willis in the SAH group rats (Additional file: Figure S1A). There was no statistical difference in SAH grading scores among the SAH groups. (Additional file: Figure S1B).

#### Time course of endogenous expressions of PEDF, 67LR, and cellular location of 67LR in the brain after SAH

**Fig. 1:**
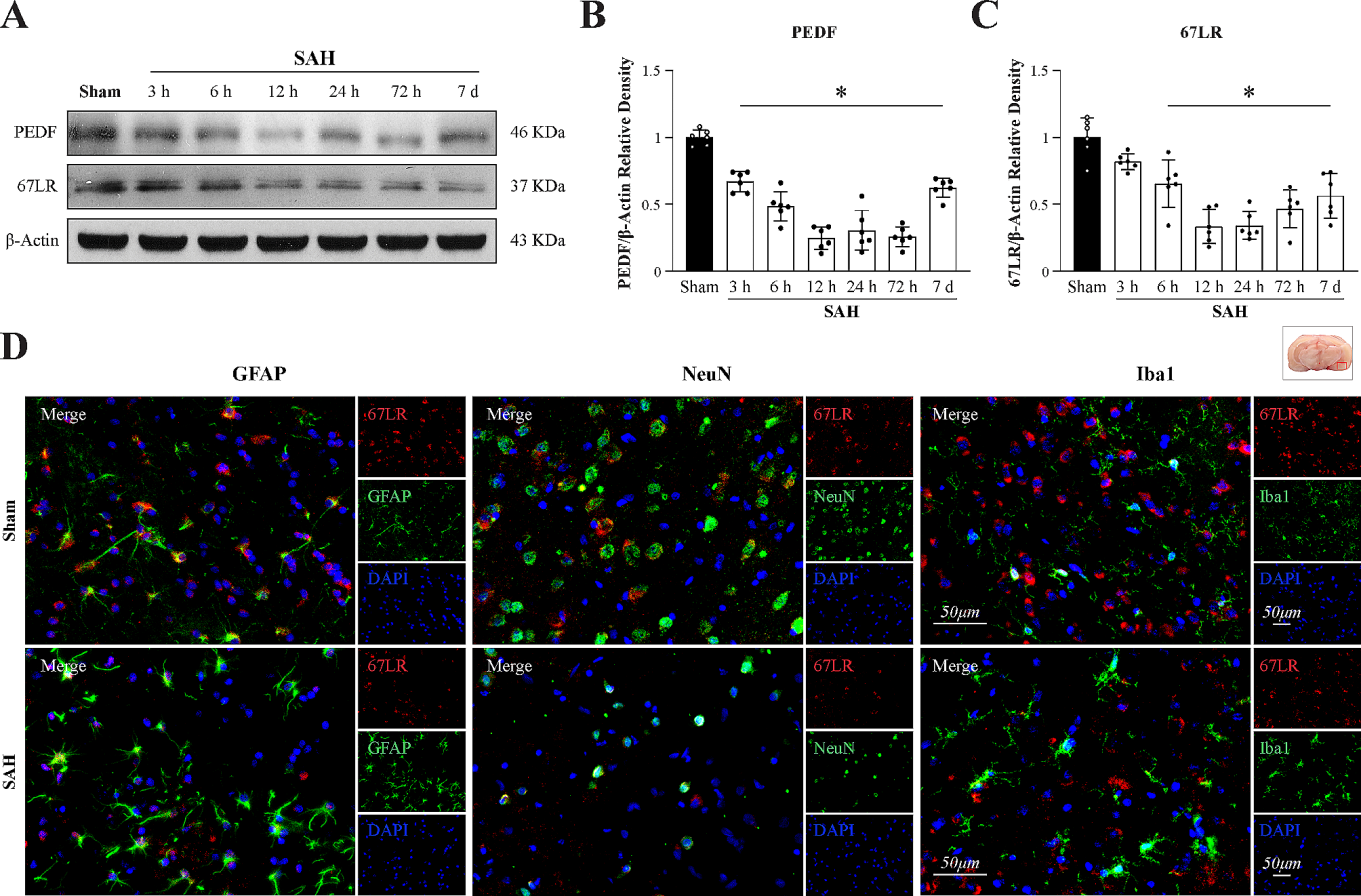
Time course of endogenous PEDF and 67LR expressions and cellular location of 67LR in the brain after SAH. (**a-c**) Representative western blot images and quantitative analyses of endogenous PEDF, 67LR expression at 3 h, 6 h, 12 h, 24 h, 72 h, and 7 d after SAH. ^*^p < 0.05 vs. Sham, *n* = 6 /group. (**d**) Representative microphotographs of immunofluorescence staining showing the colocalization of 67LR (red) with astrocyte (GFAP, green, left), neuron (NeuN, green, middle), or microglia (Iba1, green, right) in sham and SAH rats at 24 h after SAH. A small red box in the brain block indicated where the microphotographs were taken. Scale bar = 50 μm. *n* = 4/group

There was a significant decrease in the PEDF level starting at 3 h after SAH compared to the Sham group, and the level reached the lowest at 12 h after SAH (Fig. 1a, b). The 67LR level significantly started decreasing at 6 h and reached the lowest at 24 h after SAH (Fig. 1a, c). Furthermore, immunofluorescence staining showed that 67LR was expressed on astrocytes and neurons but was barely observed on microglia in the ipsilateral basal cortex at 24 h after sham surgery and SAH (Fig. 1d).

#### Intranasal administration of PEDF-34 ameliorated short-term neurological deficits at 24 h after SAH

**Fig. 2:**
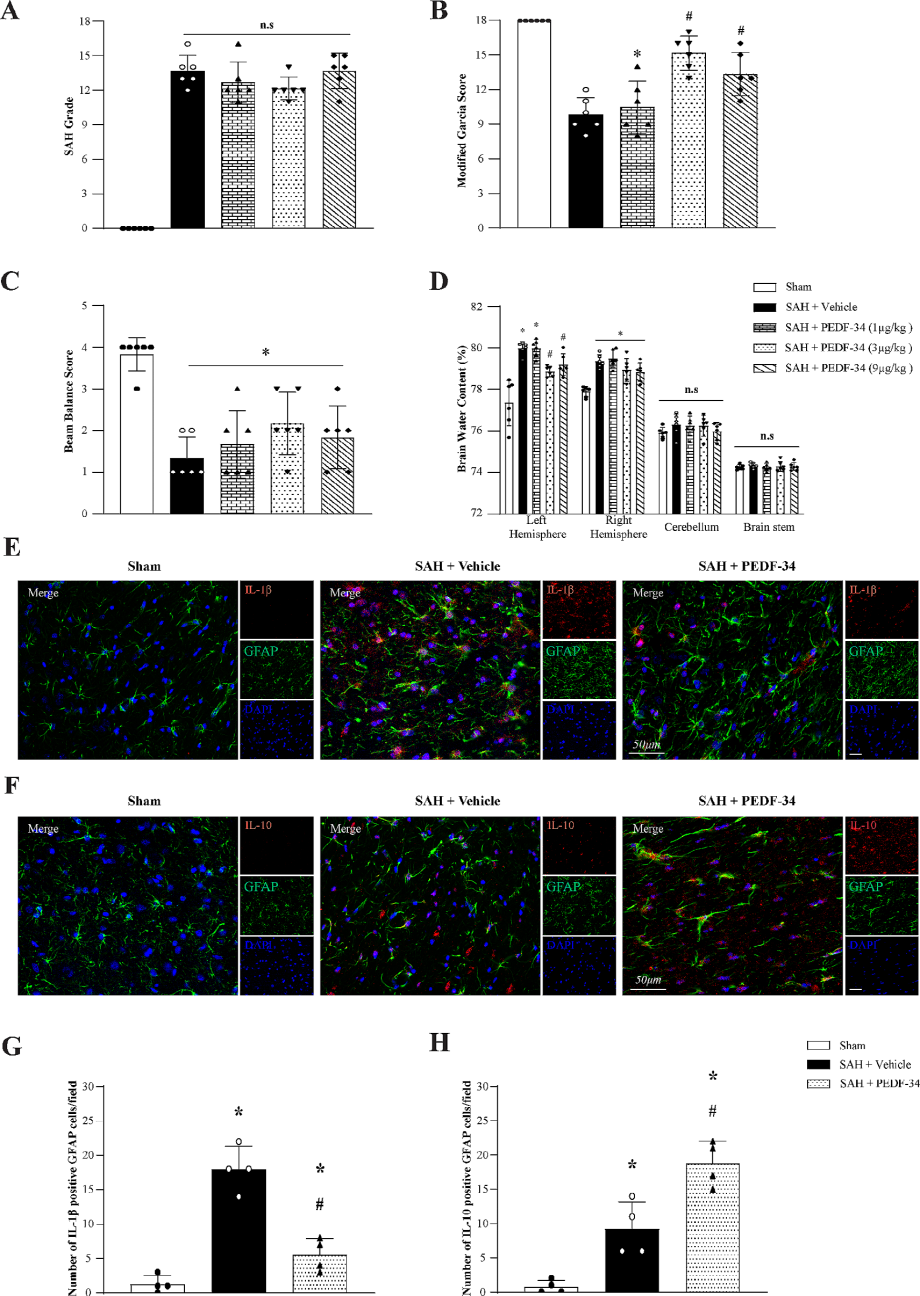
The effects of PEDF-34 on short-term outcomes at 24 h after SAH. (**a**) SAH grading scores. (**b-c**) Modified Garcia and beam balance test. ^*^p < 0.05 vs. Sham; ^#^*p* < 0.05 vs. SAH + Vehicle. (**d**) Quantitative analysis of brain water content in the left/right hemisphere, cerebellum, and brain stem. *n* = 6 /group. ^*^p < 0.05 vs. Sham; ^#^*p* < 0.05 vs. SAH + Vehicle. (**e**) Representative microphotographs of IL-1β (red) and GFAP (green) immunofluorescence staining in the ipsilateral basal cortex. Nuclei were stained with DAPI (blue). Scale bar = 50 μm. (**f**) Representative immunofluorescence images of IL-10 (red) and GFAP (green) expression in the ipsilateral basal cortex of different groups. Scale bar = 50 μm. (**g**) Quantitative analysis of IL-1β-positive astrocytes. (**h**) Quantitative analysis of IL-10-positive astrocytes. *n* = 4 /group. ^*^p < 0.05 vs. Sham; ^#^*p* < 0.05 vs. SAH + Vehicle

There was no significant difference in SAH grades among the SAH groups (Fig. 2a). The modified Garcia score and beam balance score were lower in SAH + Vehicle and SAH + PEDF-34 (1 µg/kg) groups than in the Sham group but were significantly improved in SAH rats treated with PEDF-34 at the dose of 3 µg/kg or 9 µg/kg (Fig. 2b, c). The brain water content of both cerebral hemispheres was significantly higher in the SAH + Vehicle and SAH + PEDF-34 (1 µg/kg) groups compared to the sham group (Fig. 2d), while the middle dose (3 µg/kg) and high dose (9 µg/kg) of PEDF-34 treatment significantly reduced brain water contents (Fig. 2d) in SAH rats. However, the middle-dose group showed better performance than the high-dose group. Based on the findings, the optimal dose of PEDF-34 was determined as 3 µg/kg and used in the subsequent experiments.

Immunofluorescence staining showed IL-1β and IL-10-positive astrocytes significantly increased in the SAH + Vehicle group compared with the Sham group (Fig. 2e-h). However, PEDF-34 treatment significantly reduced IL-1β-positive astrocytes (Fig. 2e, g) while increasing IL-10-positive astrocytes (Fig. 2f, h).

### PEDF-34 ameliorated long-term neurological dysfunction after SAH

**Fig. 3:**
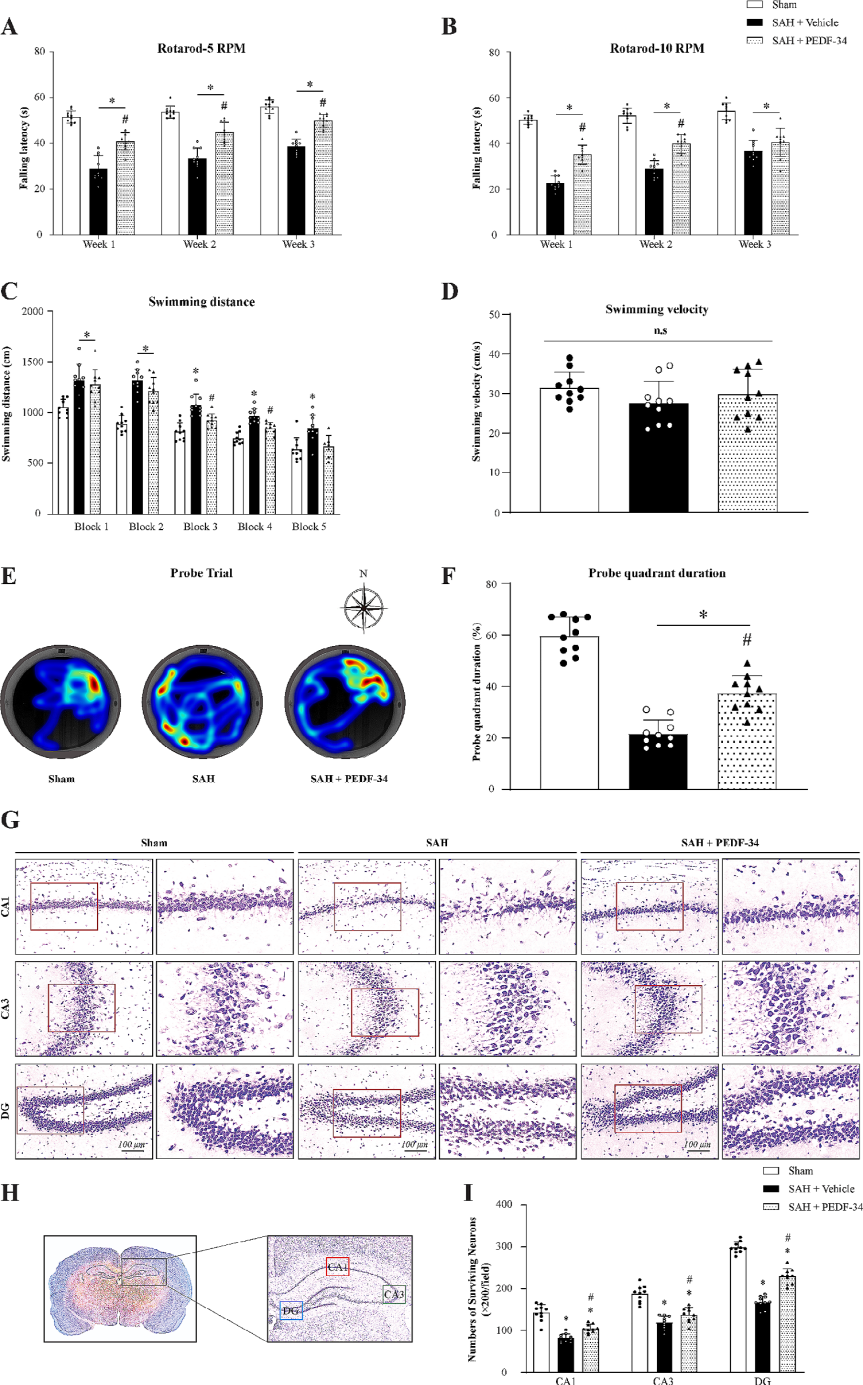
PEDF-34 reduced neuronal loss in the hippocampus and improved long-term neurological function after SAH. **(a**,** b)** The falling latency of rotarod test at 1 week, 2 weeks and 3 weeks after SAH. (**c**) Swimming distance of Morris water maze test on days 23 to 27 after SAH. (**d**) Quantification of swimming velocities. (**e-f**) Representative thermal images of the probe trials and probe quadrant duration. *n* = 10/group, ^*^p < 0.05 vs. Sham; ^#^*p* < 0.05 vs. SAH + Vehicle. (**g-h**) Representative microphotographs of Nissl staining in CA1, CA3, and DG hippocampal regions. Scale bar = 100 μm. Small red squares indicated the area for high magnification and quantification. (**i**) Quantitative analysis of surviving neuron number in different hippocampal regions. *n* = 10/group. ^*^p < 0.05 vs. Sham; ^#^*p* < 0.05 vs. SAH + Vehicle

To evaluate the effect of PEDF-34 on long-term neurological function, Rotarod and Morris water maze tests were performed after SAH. The Rotarod test showed that the latency to fall of SAH + Vehicle treated group was significantly reduced in both 5RPM and 10RPM-accelerating velocity tests compared to the sham group at 1 and 2 weeks after SAH (Fig. 3a, b). However, treatment of PEDF-34 significantly prolonged the fall latency time compared with SAH + Vehicle group after SAH (Fig. 3a, b).

In the water maze test, there was no significant difference in the swimming distance among all the groups in the block 1 and 2 tests. From block 3 to block 5 tests, there were time-related reductions in swimming distance in the sham and SAH + PEDF-34 groups. However, no significant improvement was observed in the SAH + Vehicle group (Fig. 3c). During the same period, the groups had no significant difference in swimming velocity (Fig. 3d). The probe trial showed that the SAH + PEDF-34 group spent a significantly longer time in the quadrants than the SAH + Vehicle group (Fig. 3e, f). In addition, Nissl staining showed that in the SAH + Vehicle group, compared with the sham-operated group, neurons in the CA1, CA3, and dentate gyrus (DG) areas of the ipsilateral hippocampus were significantly lost 28 days after SAH (Fig. 3g-i). However, neuronal damage was significantly reduced in the PEDF-34-treated group compared with the Vehicle group (Fig. 3i).

#### PEDF-34 inhibit astrocyte A1 polarization via 67LR/JNK/STAT1 signaling pathway

**Fig. 4:**
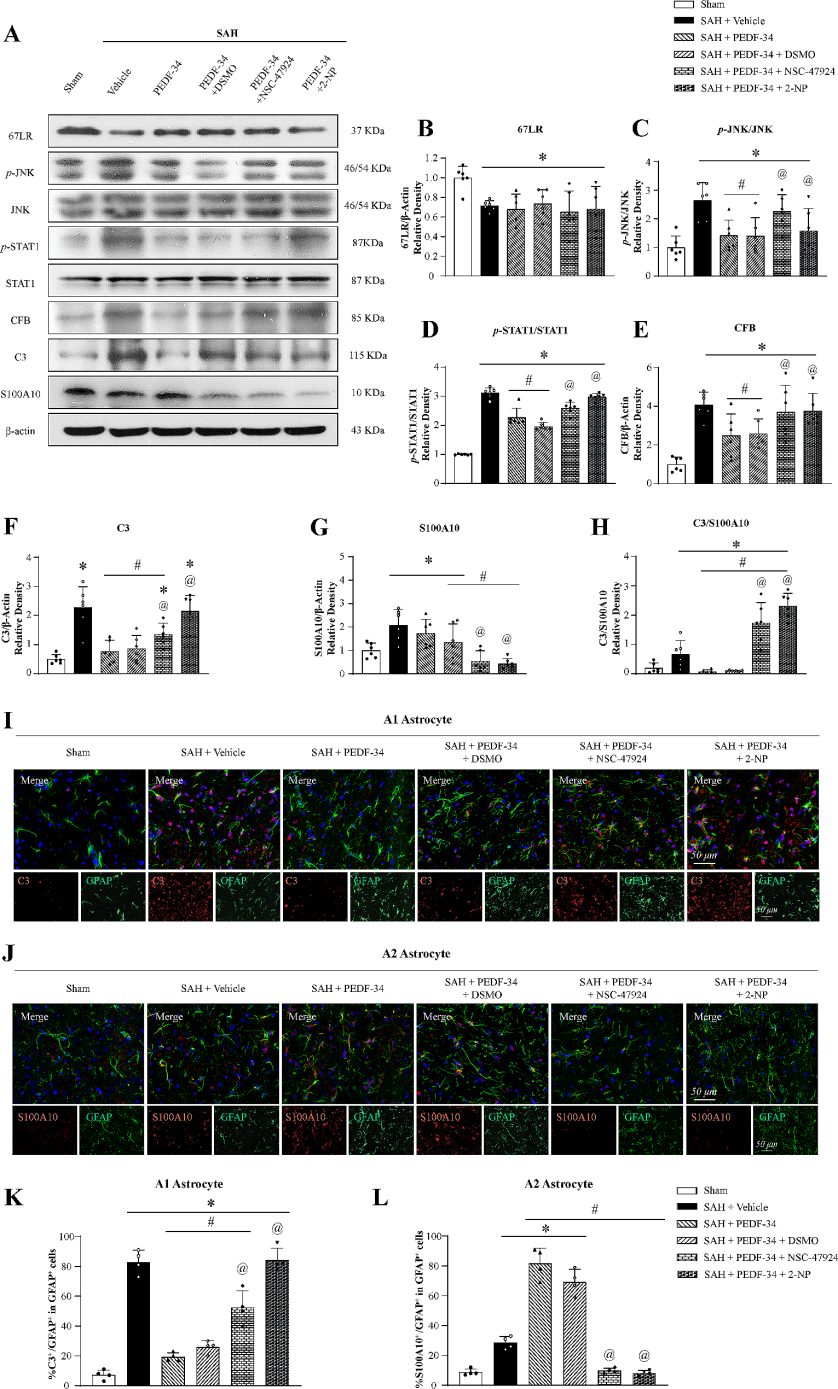
PEDF-34 promotes A2 astrocyte polarization via 67LR/JNK/STAT1 signaling pathway after SAH. (**a**) Representative western blotting bands. (**b-h**) quantitively analysis of 67LR, *p*-JNK/JNK, *p*-STAT1/STAT1, CFB, C3, S100A10 expression. *n* = 6/ group. ^*^p < 0.05 vs. Sham; ^#^*p* < 0.05 vs. SAH + Vehicle, ^@^*p* < 0.05 vs. SAH + PEDF-34 or SAH + PEDF-34 + DMSO. (**i**) Representative microphotographs of C3 (A1 Astrocyte marker, red) and GFAP (green) by immunofluorescent staining. Scale bar = 50 μm. (**k**) Quantification of C3-positive A1 astrocytes. *n* = 4/group. ^*^p < 0.05 vs. Sham; ^#^*p* < 0.05 vs. SAH + Vehicle, ^@^*p* < 0.05 vs. SAH + PEDF-34 or SAH + PEDF-34 + DMSO. (**j**) Representative microphotographs of S100A10 (A2 Astrocyte marker, red) and GFAP (green) immunofluorescence staining. Scale bar = 50 μm. (**l**) Quantification of S100A10-positive A2 astrocytes. *n* = 4/group. ^*^p < 0.05 vs. Sham; ^#^*p* < 0.05 vs. SAH + Vehicle, ^@^*p* < 0.05 vs. SAH + PEDF-34 or SAH + PEDF-34 + DMSO

To explore the underlying mechanism of PEDF to protect the brain against hemorrhage after SAH, 67LR specific inhibitor, NSC-47924, and STAT1 transcriptional activator, 2-NP was administrated intracerebroventricularly 48 h before SAH induction. Western blot results showed that 67LR expression was significantly decreased. In contrast, the expression of *p*-JNK, *p*-STAT1, CFB (A1 astrocyte marker), C3 (A1 astrocyte marker), S100A10 (A2 astrocyte marker) and the ratio of C3/S100A10 was significantly increased at 24 h after SAH compared with the sham group (Fig. 4a-h). PEDF-34 treatment decreased expressions of *p*-JNK, *p*-STAT1, CFB, and the ratio of C3 to S100A10 (Fig. 4a-g). 67LR inhibitor, NSC-47924, did not affect 67LR expressions (Fig. 4b-g) but reversed the effects of PEDF-34 on expressions of *p*-JNK, *p*-STAT1, CFB and C3 (Fig. 4b-g). Furthermore, the STAT1 transcriptional activator (2-NP) reversed the effects of PEDE-34 treatment on *p*-STAT1, CFB, and C3 expression in SAH rats (Fig. 4b-h).

The effects of 67LR on astrocytic polarization were assessed by immunofluorescence staining. C3 (Fig. 4i) and S100A10 (Fig. 4j) expressions on astrocytes were increased after SAH. While in PEDF-34 treated SAH rats, there was a remarkable decrease in C3-positive astrocytes (Fig. 4i, k), but S100A10-positive astrocytes were further significantly increased (Fig. 4j, l) compared to the SAH + Vehicle group. However, the effects of PEDF-34 on astrocyte phenotype changes were reversed by treatment of NSC-47924 or 2-NP (Fig. 4i-l).

#### Effects of PEDF-34 administration on JNK/STAT1 signaling pathway and A1/A2 astrocyte polarization in Hb-stimulated primary astrocytes

**Fig. 5:**
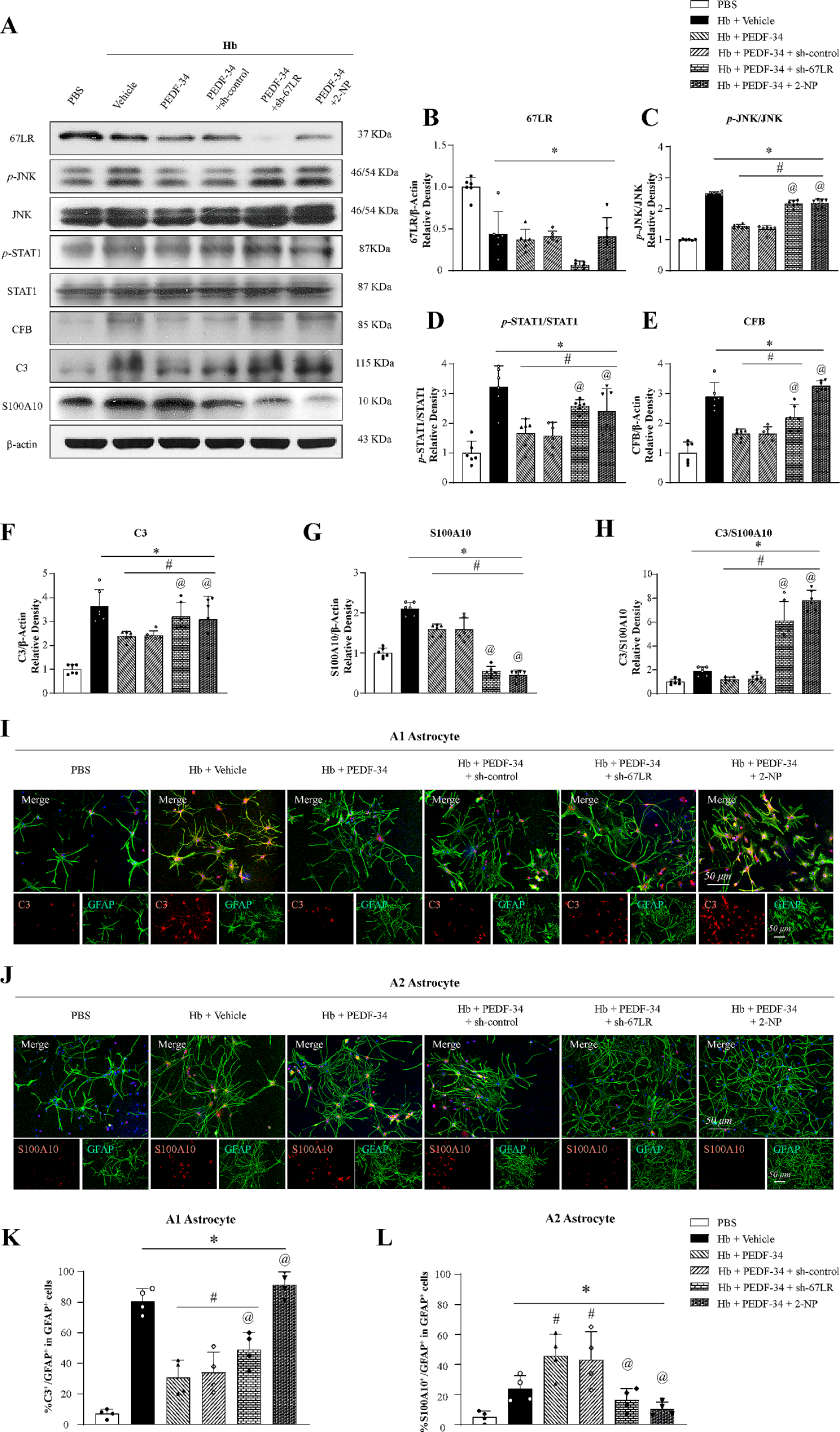
PEDF-34 modulated astrocyte polarization by 67LR/JNK/STAT1 signaling pathway in vitro. (**a**) Representative western blotting and (**b-h**) quantitively analysis of 67LR, *p*-JNK/JNK, *p*-STAT1/STAT1, CFB, C3 and S100A10 expressions. *n* = 6/group. ^*^p < 0.05 vs. PBS; ^#^*p* < 0.05 vs. Hb + Vehicle, ^@^*p* < 0.05 vs. Hb + PEDF-34 or Hb + PEDF-34 + sh-control. (**i**) Representative microphotograph images of colocalization of C3 (A1 marker, red) and GFAP (green) immunofluorescence staining. Scale bar = 50 μm. (**k**) Quantification of C3-positive A1 astrocyte. *n* = 4/group. ^*^p < 0.05 vs. PBS; ^#^*p* < 0.05 vs. Hb + Vehicle, ^@^*p* < 0.05 vs. Hb + PEDF-34 or Hb + PEDF-34 + sh-control. PEDF-34 effect on A2 astrocytes at 24 h after Hb stimulation. (**j**) Representative microphotographs of S100A10 (A2 marker, red) and GFAP (green) immunofluorescence staining. Scale bar = 50 μm. (**l**) Quantification of S100A10 positive A2 astrocytes. *n* = 4 replicates/group. ^*^p < 0.05 vs. PBS; ^#^*p* < 0.05 vs. Hb + Vehicle, ^@^*p* < 0.05 vs. Hb + PEDF-34 or Hb + PEDF-34 + sh-control

Primary astrocyte culture experiment was performed to verify whether the effects of PEDF was mediated by modulating astrocyte polarization through JNK/STAT1 signaling pathway. The results showed that Hb stimulation significantly decreased 67LR expressions but increased *p*-JNK, *p*-STAT1, CFB, C3, and S100A10 (Fig. 5a-h). Compared with the Hb + Vehicle group, expressions of p-JNK, p-STAT1, CFB, and C3 were decreased, while the expression of S100A10 was increased in the Hb + PEDF-34 treated group (Fig. 5a-h).

Knockdown of 67LR with 67LR shRNA significantly decreased the 67LR expression and reversed the effects of PEDF-34 on *p*-JNK, *p*-STAT1, CFB and C3 expressions compared with Hb + PEDF34 or Hb + PEDF-34 + sh-control group (Fig. 5a-g). 2-NP reversed the effect of PEDF-34 treatment on expressions of *p*-STAT1, CFB, C3 and S100A10 compared to Hb + PEDF-34 or Hb + PEDF-34 + sh-control group (Fig. 5a-g). Immunofluorescence staining consistently showed that PEDF-34 decreased C3 positive astrocytes (Fig. 5i, k) but increased S100A10 positive astrocytes at 24 h after Hb stimulation compared to Hb + Vehicle group or Hb + PEDF-34 + sh-control (Fig. 5j, l). These effects were reversed by knockdown of 67LR or STAT1 transcriptional activator treatment (Fig. 5i-l).

### PEDF-34 inhibits astrocyte-mediated inflammatory cytokines secretion through 67LR/JNK/STAT1 signaling pathway in vivo and in vitro

**Fig. 6:**
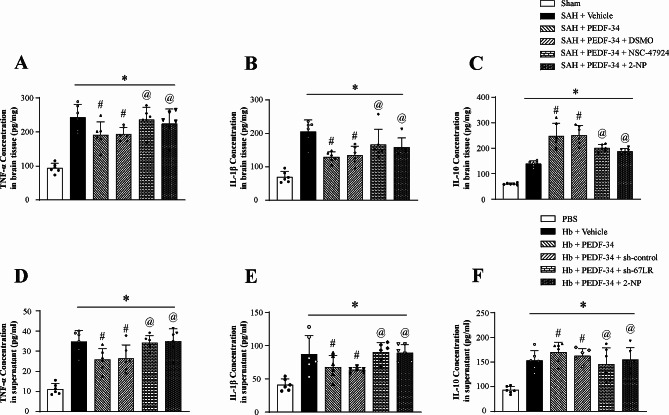
PEDF-34 attenuated astrocytic pro-inflammatory cytokines in vivo and in vitro. (**a-c**) ELISA analysis of TNF-α, IL-1β, and IL-10 level. *n* = 6/group. ^*^p < 0.05 vs. Sham; ^#^*p* < 0.05 vs. SAH + Vehicle, ^@^*p* < 0.05 vs. SAH + PEDF-34 or SAH + PEDF-34 + DMSO. (**d-f**) ELISA analysis of TNF-α, IL-1β, and IL-10 levels in astrocyte culture medium. *n* = 4/group. ^*^p < 0.05 vs. PBS; ^#^*p* < 0.05 vs. Hb + Vehicle, ^@^*p* < 0.05 vs. Hb + PEDF-34 or Hb + PEDF-34 + sh-control

ELISA results showed that TNF-α, IL-1β, and IL-10 were significantly elevated at 24 h after SAH. PEDF-34 treatment reduced the level of pro-inflammatory cytokines TNF-α (Fig. 6a) and IL-1β (Fig. 6b) but elevated the level of anti-inflammatory cytokine IL-10 (Fig. 6c) in SAH rats. Intraventricular administration of 67LR inhibitor, NSC-47924 and the STAT1 transcriptional activator, 2-NP, reversed the effect of PEDF-34 on the secretion of inflammatory relative cytokines (Fig. 6a-c).

In in vitro study, ELISA results showed that TNF-α, IL-1β and IL-10 levels in the astrocytes culture medium significantly increased at 24 h after Hb stimulation (Fig. 6d-f). Incubation of PEDF-34 reduced the level of pro-inflammatory cytokines of TNF-α (Fig. 6d) and IL-1β (Fig. 6e) but further elevated the level of anti-inflammatory cytokine IL-10 (Fig. 6f). Knock-down of 67LR with Sh-67LR or activation of STAT1 with 2-NP reverses the effect of PEDF-34 on TNF-α, IL-1β and IL-10 secretion (Fig. 6d-f).

## Discussion

In the present study, we found that the endogenous protein of PEDF and 67LR was decreased after SAH in rats. 67LR were expressed in astrocytes and neurons but barely in microglia. The activation of 67LR with PEDF-34 significantly improved the short-term neurobehavioral deficits and attenuated brain edema, accompanied by decreased pro-inflammatory cytokines IL-1β and IL-10 at 24 h after SAH. In addition, PEDF-34 improved long-term neurobehavioral function 4 weeks after SAH. Mechanistically, PEDF-34 treatment suppresses the ratio of *p*-JNK/JNK, *p*-STAT1/STAT1, CFB, and C3 expression but increases the expression of S100A10. Furthermore, the expression of *p*-JNK/JNK, *p*-STAT1/STAT1, CFB, C3, and S100A10 was associated with SAH-induced PEDF and 67LR expression changes (Additional file: Figure S3A). 67LR specific inhibitor, NSC-47924/ knockdown of 67LR with Sh-67LR or STAT1 transcriptional activator, 2-NP abolished the beneficial effects of PEDF-34 on A1/A2 astrocyte polarization and astrocyte-mediated neuroinflammation in vivo and in vitro. Furthermore, we found that PEDF-34 alleviates delayed neuronal degeneration in the hippocampus after SAH. Taken together, our findings suggest that PEDF-34 could activate 67LR, promote A2 astrocyte polarization, and suppress astrocyte-mediated neuroinflammation, partially by JNK/STAT1 signaling pathway.

Pigment Epithelium-Derived Factor, PEDF, was first found to be secreted by the retinal pigment epithelium in the eye, after which it was detected in both neurons and glia of the brain [5, 37]. Previous studies demonstrated that PEDF was neuroprotective through its neurotrophic effect, anti-inflammation, anti-oxidative stress, and preservation of BBB integrity [11, 38–40]. Upon activation of 67LR, PEDF mitigates the formation of status epilepticus-induced vasogenic edema by inhibiting the p38 MAPK–PI3K/AKT/eNOS axis [18]. 67LR activations have been found to promote functional recovery after stroke and spinal cord injury [41, 42]. However, whether PEDF/67LR signaling plays a role in SAH remains unclear.

To investigate the protective effect of PEDF in SAH, PEDF 34-mer peptide (PEDF-34) was used in the experiments. PEDF-34, derived from amino acid positions Asp44–Asn77 of the full-length PEDF, exhibits a higher half-life, production yield, purity, and smaller size than the full-length PEDF. These characteristics ensure the stable functioning of PEDF-34 within the brain [11, 43, 44]. Our in vivo study demonstrated that SAH rats treated with PEDF-34 at the dose of 3 µg/kg exhibited significant improvements in short-term neurobehavioral deficits 24 h after SAH. Moreover, PEDF-34 improved long-term outcomes, including on the rotarod and water maze tests in SAH rats. Better spatial learning function was associated with less neuronal damage within ipsilateral hippocampal CA1, CA3, and dentate gyrus region.

67LR activations inhibit inflammatory mediators and cytokine levels in various cells, such as cerebral microvascular endothelial cells, macrophages, and adipocytes [45–48]. A recent study demonstrated that 67LR activation by Epigallocatechin gallate (EGCG) inhibited endotoxin-induced expression of inflammatory cytokines, including TNF-α and IL-1β, in human cerebral microvascular endothelial cells [46]. After SAH, the level of pro-inflammatory cytokines TNF-a and IL-1β increased in the brain tissue [49, 50]. Consistently, pro-inflammatory cytokines were higher in the brains of SAH rats but decreased after PEDF-34 treatment. However, inhibition of 67LR reversed the anti-inflammatory effects of PEDF-34, suggesting 67LR mediates the neuroprotective effects of PEDF-34.

Reactive astrocytes play an essential role in regulating neuroinflammation during EBI after SAH. In response to SAH, astrocytes undergo reactive changes and are further classified into harmful A1 phenotype and protective A2 phenotype, contributing to neuronal death or upregulating specific neurotrophic factors for neuronal survival, respectively [45]. Specifically, active A1 astrocytes induce neuronal apoptosis by releasing pro-inflammatory factors after SAH [25]. Consistently in the current study, the number of A1 phenotype astrocytes significantly increased in SAH rats and primary astrocytes stimulated by Hb. Both in vivo and in vitro experiments showed that PEDF-34 reduced A1 phenotype astrocytes and increased A2 phenotype astrocytes after SAH. However, the therapeutic effects of PEDF-34 were partially reversed by inhibiting 67LR with NSC-47924 or knocking down 67LR with 67LR shRNA. These findings suggest that activation of PEDF-34/67LR signaling promotes astrocytic A1 to A2 phenotype changes, resulting in decreased secretion of pro-inflammatory cytokines and increased anti-inflammatory cytokine secretion in the brain after SAH.

The phosphorylation of JNK and STAT1 can increase A1 type astrocytes number and activation of the complement system, resulting in a solid inflammatory effect [3, 51]. In the current study, the phosphorylation level of JNK and STAT1 significantly increased in rat brains after SAH and in Hb-stimulated primary astrocytes, while PEDF-34 decreased the phosphorylation level of JNK and STAT1. However, 67LR agonist, NSC-47924, or knockdown with 67LR shRNA reversed the effect of PEDF-34 in vivo and in vitro, respectively. Changes in the phosphorylation level of JNK and STAT1 are associated with 67LR activation and inhibition, suggesting that JNK and STAT1 were the downstream molecules of this signaling pathway.

However, the study had limitations. Firstly, we focused on PEDF/67LR’s effect on astrocyte-mediated neuroinflammation. However, 67LR is also expressed in neurons; the PEDF-34/67LR-related neuronal protective mechanisms require further investigation. Secondly, patients with clinically poor prognosis after SAH have significantly elevated PEDF levels in the acute phase of plasma [52]. In contrast, in our study, there was a significant decrease in endogenous PEDF and 67LR expression in the ipsilateral brain at the early stage after experimental SAH, accompanied by increased *p*-JNK and *p*-STAT1 expressions and ratio of A1/A2 astrocytes phenotypes. Further studies on the expression of PEDF in the brains of SAH patients are needed, and the clinical role of PEDF needs to be further explored. Lastly, gender differences and age effects were not addressed in the current study.

## Conclusions

Our results demonstrated that 67LR activation by PEDF-34 promoted astrocyte A2 polarization via the JNK/STAT1 pathway, thus attenuating neuroinflammation and improving neurological outcomes after experimental SAH. These findings suggested that early administration of PEDF-34 might serve as a promising therapeutic approach for EBI and delayed brain injury following SAH.

### Electronic supplementary material

Below is the link to the electronic supplementary material.

Supplementary Material 1

Supplementary Material 2

Supplementary Material 3

Supplementary Material 4

## Data Availability

No datasets were generated or analysed during the current study.

## Abbreviations

SAH: Subarachnoid hemorrhage
EBI: Early brain injury
PEDF: Pigment epithelium-derived factor
67LR: 67-kDa non-integrate laminin receptor
BBB: Blood-brain barrier
2-: NP 2-(1,8-naphthyridin-2-yl) phenol
CNS: Central nervous system
ELISA: Enzyme-linked immunosorbent assay
Iba1: Ionized calcium-binding adapter molecule 1
NeuN: Neuronal nuclei
GFAP: Glial fibrillary acidic protein
IL: Interleukin IL
shRNA: Short hairpin RNA shRNA
TNF-α: α Tumor necrosis factor alpha
AQP4: Aquaporin 4
JNK: c-Jun N-terminal kinase
CFB: Complement factor B
C3: Complement component 3
STAT1: Signal transducer and activator of transcription 1
