# Supplementary figures and images for: PEDF-34 attenuates neurological deficit and suppresses astrocyte-dependent neuroinflammation by modulating astrocyte polarization via 67LR/JNK/STAT1 signaling pathway after subarachnoid hemorrhage in rats
Source: J Neuroinflammation. 2024 Jul 21;21:178. doi: 10.1186/s12974-024-03171-y (PMC11264993; doi:10.1186/s12974-024-03171-y)

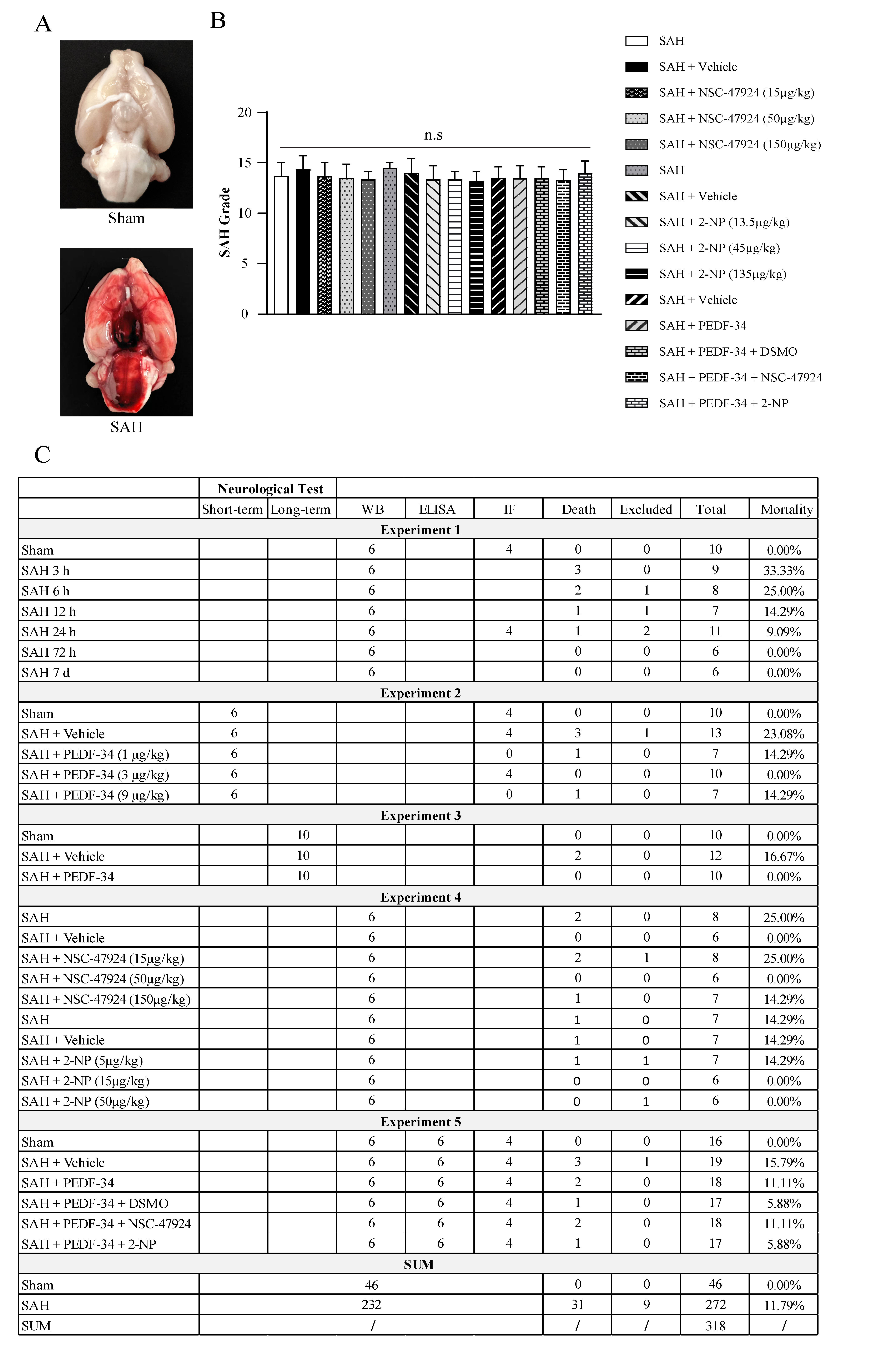

Supplement: Supplementary file 1 — Supplementary Material 1 [file 12974_2024_3171_MOESM1_ESM.tif]

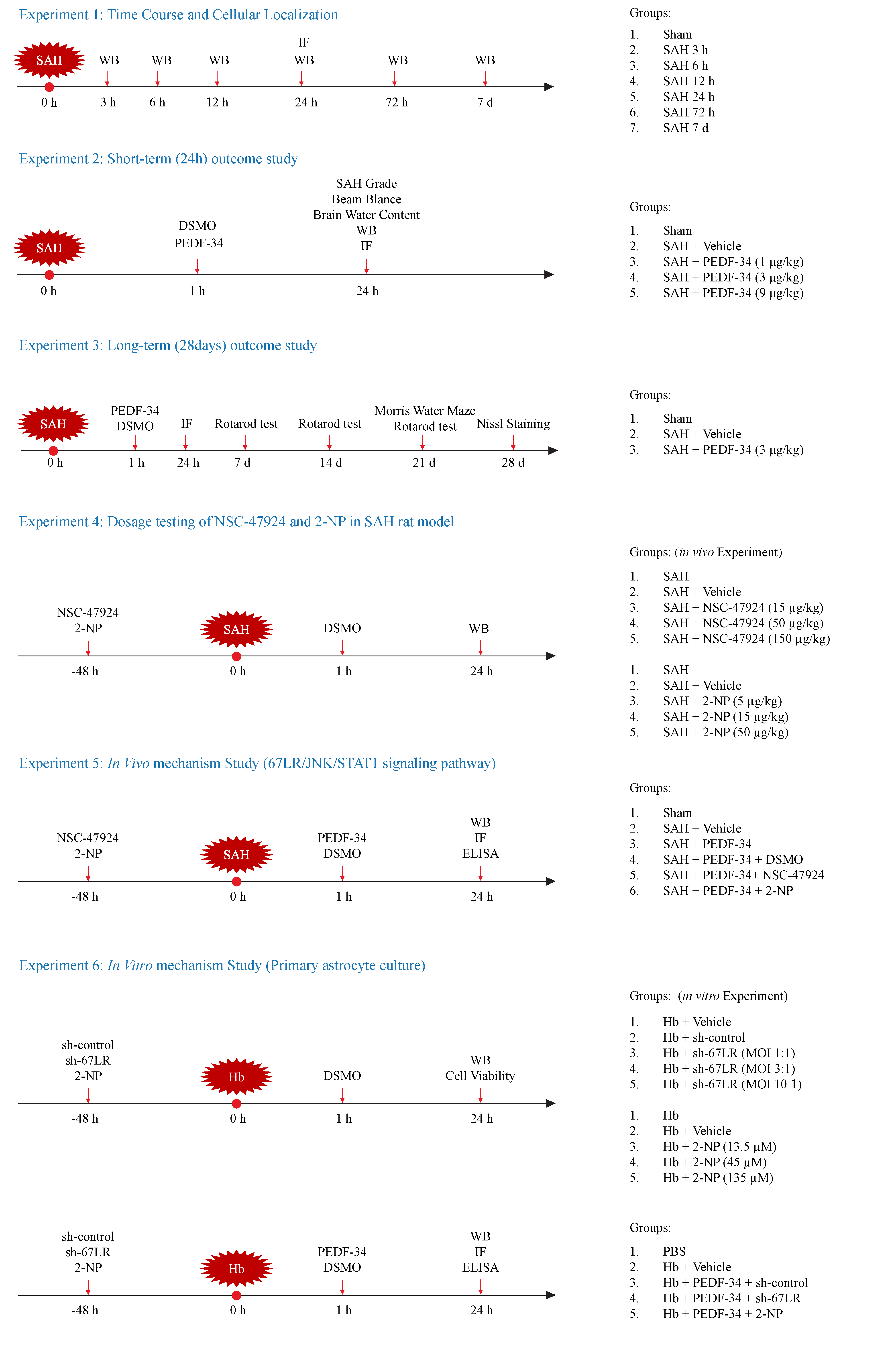

Supplement: Supplementary file 2 — Supplementary Material 2 [file 12974_2024_3171_MOESM2_ESM.tif]

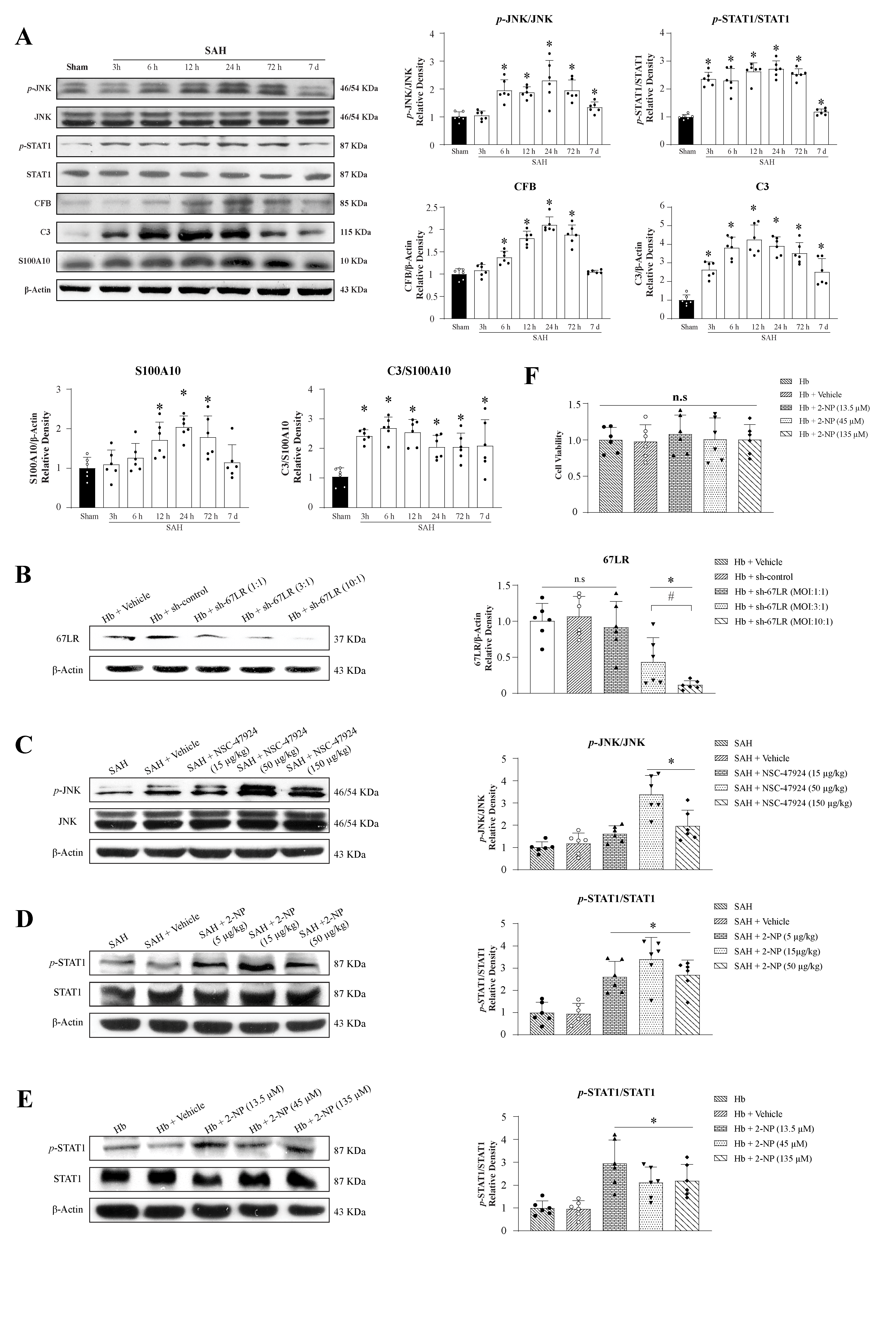

Supplement: Supplementary file 3 — Supplementary Material 3 [file 12974_2024_3171_MOESM3_ESM.tif]
