# Supplementary material for: PEDF-34 attenuates neurological deficit and suppresses astrocyte-dependent neuroinflammation by modulating astrocyte polarization via 67LR/JNK/STAT1 signaling pathway after subarachnoid hemorrhage in rats
Source: J Neuroinflammation. 2024 Jul 21;21:178. doi: 10.1186/s12974-024-03171-y (PMC11264993; doi:10.1186/s12974-024-03171-y)

Figure 1

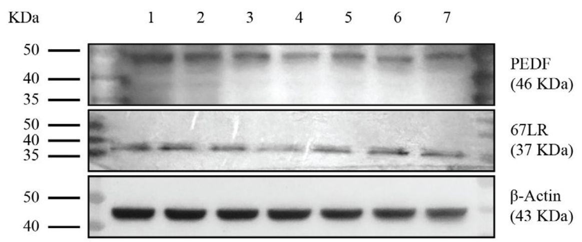

1. *Sham*
2. *SAH 3h*
3. *SAH 6h*
4. *SAH 12h*
5. *SAH 24h*
6. *SAH 72h*
7. *SAH 7d*

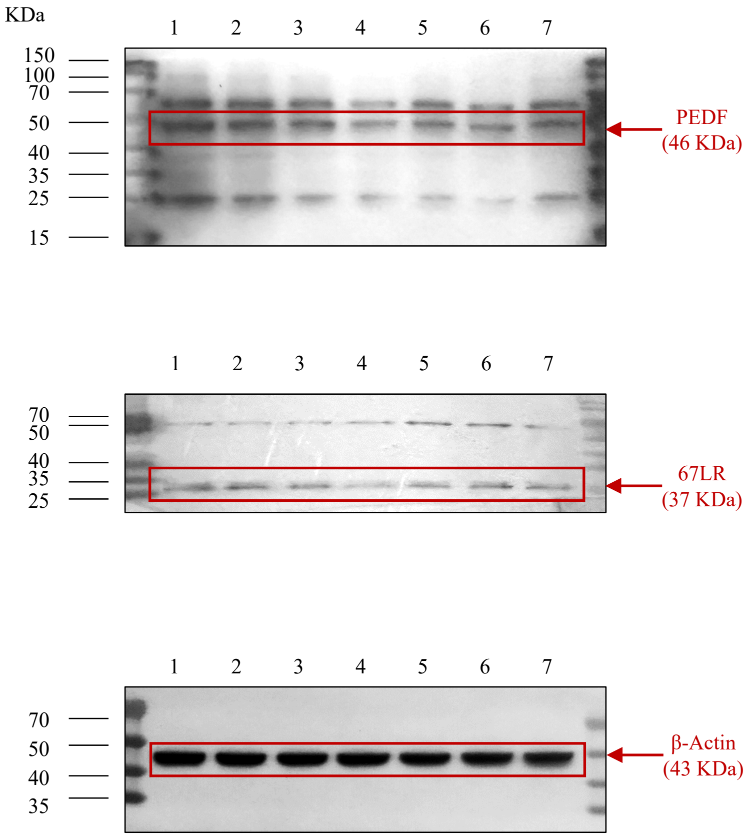

Figure 4

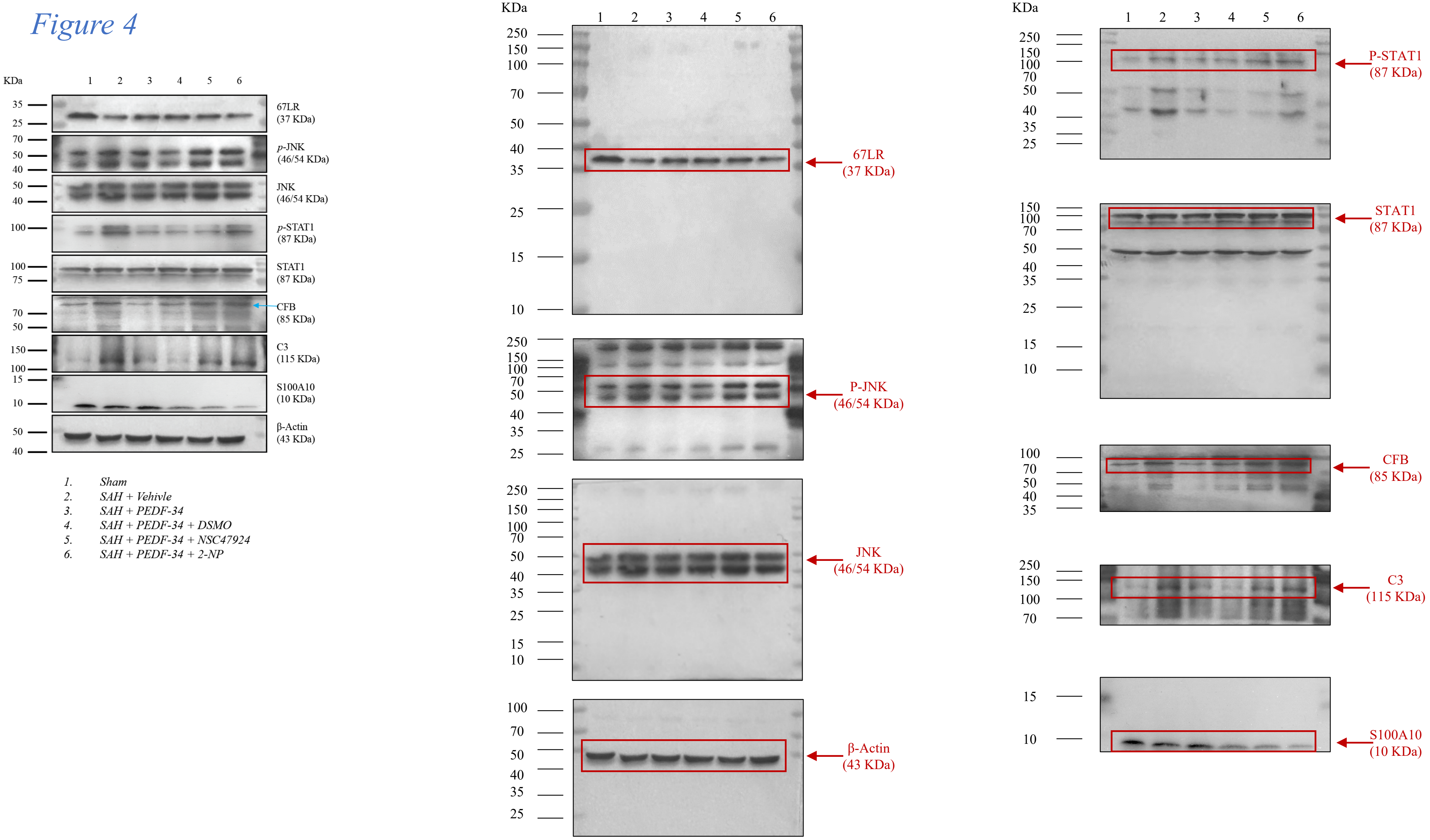
KDa
1 2 3 4 5 6
67LR (37 KDa)
p-JNK (46/54 KDa)
JNK (46/54 KDa)
p-STAT1 (87 KDa)
STAT1 (87 KDa)
CFB (85 KDa)
C3 (115 KDa)
S100A10 (10 KDa)
β-Actin (43 KDa)
1. Sham
2. SAH + Vehivle
3. SAH + PEDF-34
4. SAH + PEDF-34 + DSMO
5. SAH + PEDF-34 + NSC47924
6. SAH + PEDF-34 + 2-NP
P-JNK (46/54 KDa)
P-STAT1 (87 KDa)

Figure 5

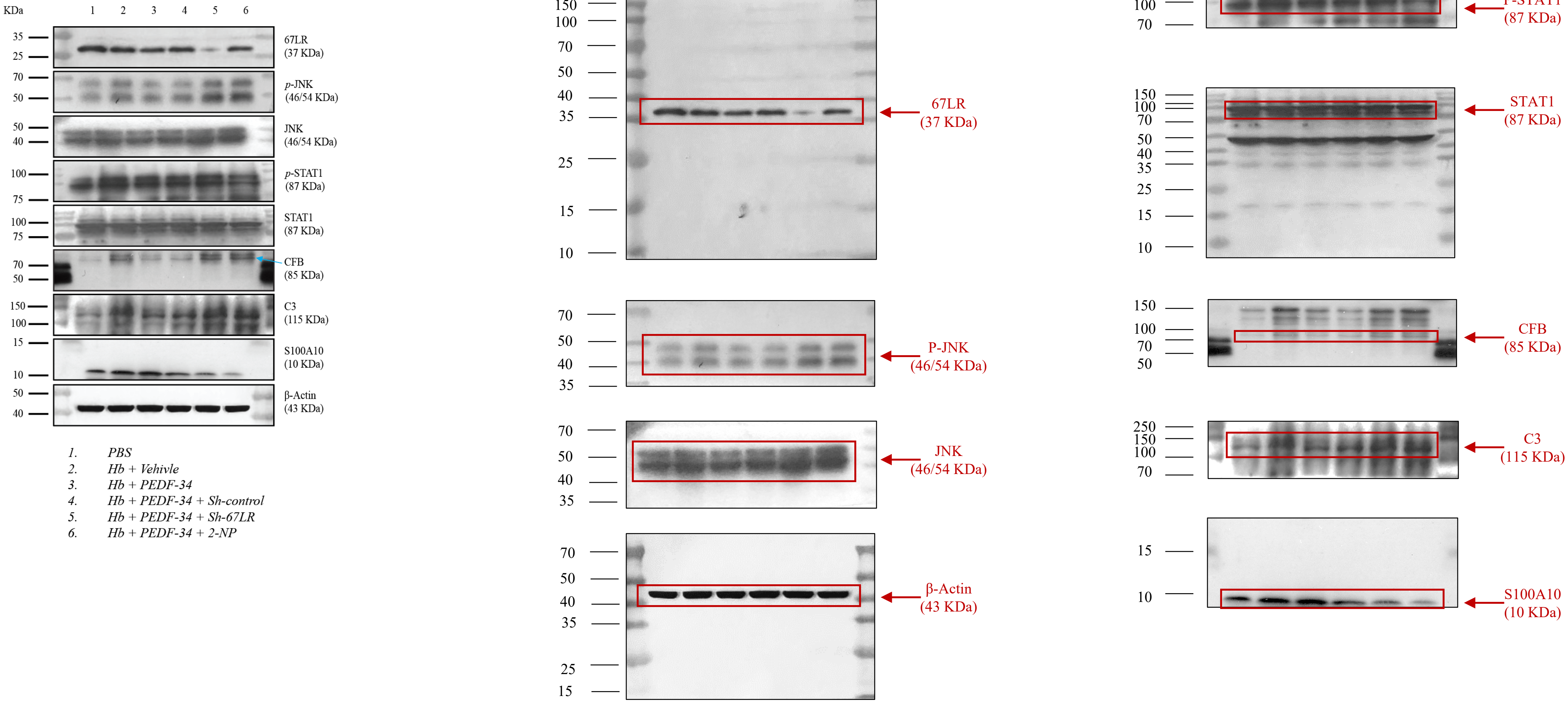
KDa
1 2 3 4 5 6
67LR (37 KDa)
p-JNK (46/54 KDa)
JNK (46/54 KDa)
p-STAT1 (87 KDa)
STAT1 (87 KDa)
CFB (85 KDa)
C3 (115 KDa)
S100A10 (10 KDa)
β-Actin (43 KDa)
1. PBS
2. Hb + Vehivle
3. Hb + PEDF-34
4. Hb + PEDF-34 + Sh-control
5. Hb + PEDF-34 + Sh-67LR
6. Hb + PEDF-34 + 2-NP
P-JNK (46/54 KDa)
P-STAT1 (87 KDa)

Figure supplementary

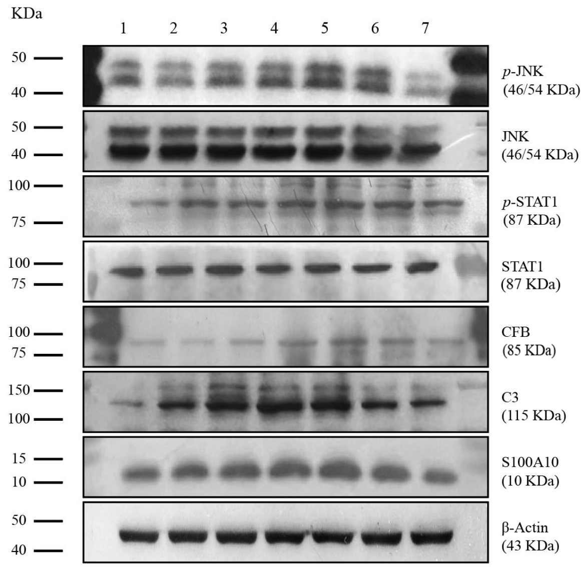

1. *Sham*
2. *SAH 3h*
3. *SAH 6h*
4. *SAH 12h*
5. *SAH 24h*
6. *SAH 72h*
7. *SAH 7d*

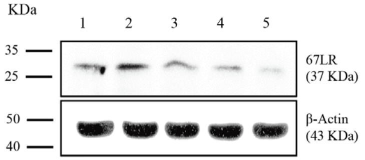

1. Hb + Vehicle
2. Hb + sh-control
3. Hb + sh-67LR (MOI 1:1)
4. Hb + sh-67LR (MOI 3:1)
5. Hb + sh-67LR (MOI 10:1)

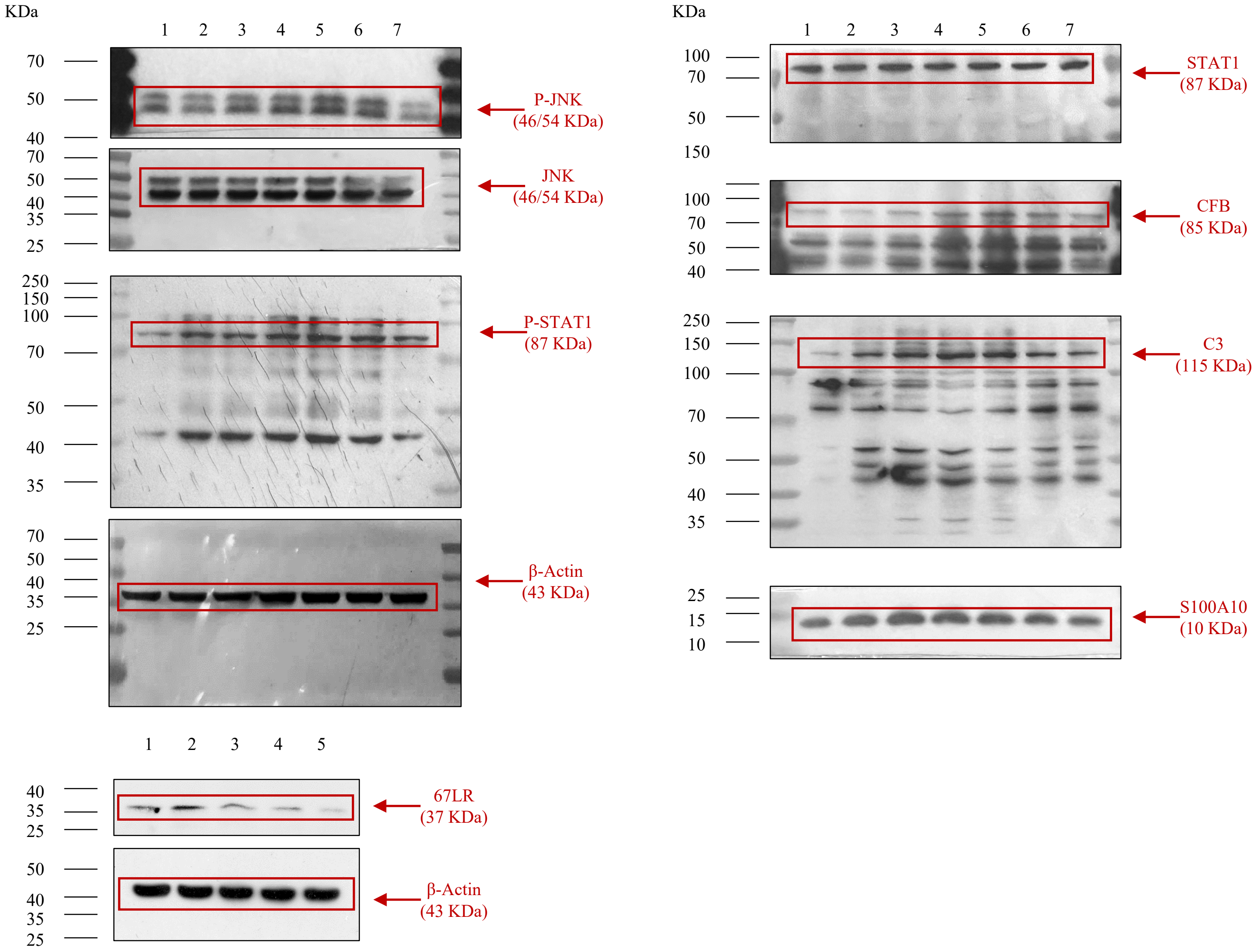

Figure supplementary

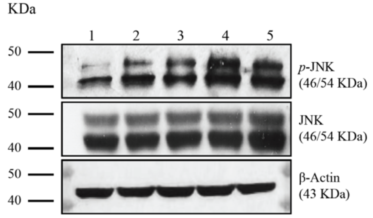

1. SAH
2. SAH + Vehicle
3. SAH + NSC-47924 (15 μg/kg)
4. SAH + NSC-47924 (50 μg/kg)
5. SAH + NSC-47924 (150 μg/kg)

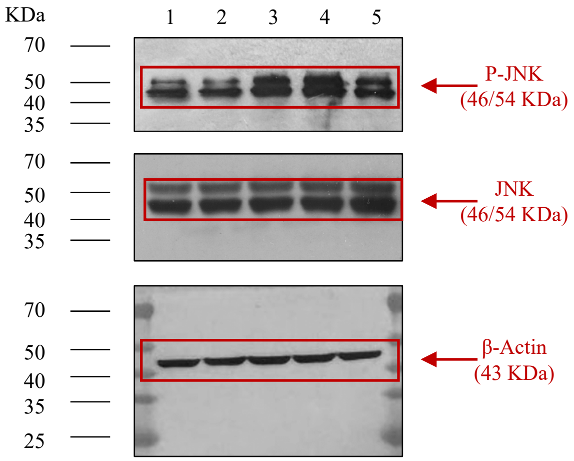

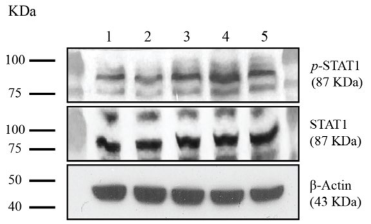

1. SAH
2. SAH + Vehicle
3. SAH + 2-NP (5 μg/kg)
4. SAH + 2-NP (15 μg/kg)
5. SAH + 2-NP (50 μg/kg)

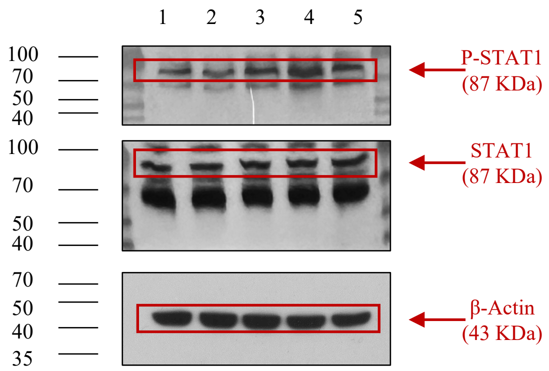

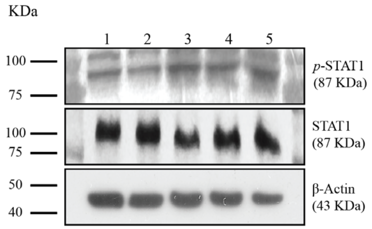

1. Hb
2. Hb + Vehicle
3. Hb + 2-NP (13.5 μM)
4. Hb + 2-NP (45 μM)
5. Hb + 2-NP (135 μM)

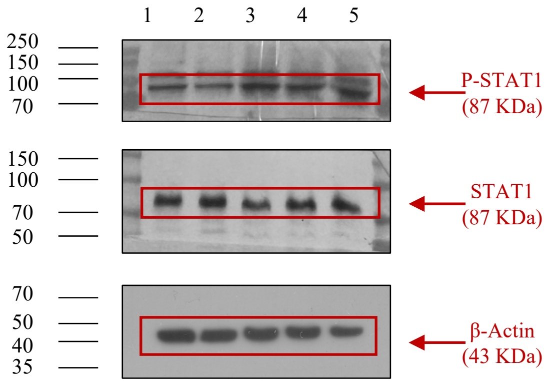

Supplement: Supplementary file 4 — Supplementary Material 4 [file 12974_2024_3171_MOESM4_ESM.pdf]
